# Simultaneous Detection and Removal of Formaldehyde at Room Temperature: Janus Au@ZnO@ZIF-8 Nanoparticles

**DOI:** 10.1007/s40820-017-0158-0

**Published:** 2017-10-09

**Authors:** Dawei Wang, Zhiwei Li, Jian Zhou, Hong Fang, Xiang He, Puru Jena, Jing-Bin Zeng, Wei-Ning Wang

**Affiliations:** 1 Department of Mechanical & Nuclear Engineering, Virginia Commonwealth University, Richmond, VA 23219 USA; 2 College of Science China University of Petroleum (East China), Qingdao, 266580 People’s Republic of China; 3 Department of Physics, Virginia Commonwealth University, Richmond, VA 23284 USA; 4 Department of Chemistry, University of California, Riverside, CA 92521 USA

**Keywords:** Indoor air quality, Volatile organic compounds, Janus structure, Metal organic frameworks, Plasmonic nanoparticles

## Abstract

The detection and removal of volatile organic compounds (VOCs) are of great importance to reduce the risk of indoor air quality concerns. This study reports the rational synthesis of a dual-functional Janus nanostructure and its feasibility for simultaneous detection and removal of VOCs. The Janus nanostructure was synthesized via an anisotropic growth method, composed of plasmonic nanoparticles, semiconductors, and metal organic frameworks (e.g., Au@ZnO@ZIF-8). It exhibits excellent selective detection to formaldehyde (HCHO, as a representative VOC) at room temperature over a wide range of concentrations (from 0.25 to 100 ppm), even in the presence of water and toluene molecules as interferences. In addition, HCHO was also found to be partially oxidized into non-toxic formic acid simultaneously with detection. The mechanism underlying this technology was unraveled by both experimental measurements and theoretical calculations: ZnO maintains the conductivity, while ZIF-8 improves the selective gas adsorption; the plasmonic effect of Au nanorods enhances the visible-light-driven photocatalysis of ZnO at room temperature.

**Electronic supplementary material:**

The online version of this article (doi:10.1007/s40820-017-0158-0) contains supplementary material, which is available to authorized users.

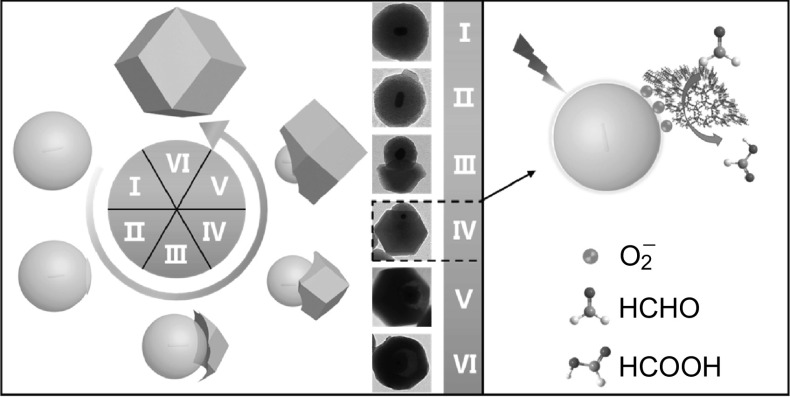

## Highlights

- Au@ZnO@ZIF-8 Janus nanostructure was designed and synthesized via an anisotropic growth method for the detection of volatile organic compounds (VOCs).
- Due to the synergistic effects of the high conductivity of ZnO, the superior gas adsorption capability of ZIF-8, the clean interface between ZnO and ZIF-8, and the plasmonic resonance of gold nanorods, the Janus nanostructure demonstrates an excellent sensing performance with a selective detection toward formaldehyde at room temperature.

## Introduction

Improved indoor air quality is essential for human health since people spend almost 90% of their life in various environments [1]. Among numerous indoor air pollutants, volatile organic compounds (VOCs) are among the most concerning ones. VOCs are omnipresent in various indoor environments, and their concentrations are consistently higher indoors than outdoors. Exposure to VOCs, typically, formaldehyde (HCHO), may cause headache, irritation, diseases, and even cancer [2]. It is thus of high interest to develop technologies for accurate detection and efficient removal of VOCs in indoor environments, which, however, has been a challenge.

The conventional gas sensors for VOCs are usually based on semiconductors. Among all the candidate semiconductors, ZnO has shown its advantages in terms of satisfactory economic efficiency, easy manipulation of the morphologies, and capacities of detecting various gases [3]. However, up to now, most of the ZnO-based sensors have to be operated at elevated temperatures, to allow the oxygen species adsorbed on the surfaces of semiconductors and initiate the reactions between them and guest molecules [4]. Further exploration of this field has thus been limited. Several strategies have been proposed to lower the operating temperature of ZnO-based sensors [5]. Among them, photo-induced gas sensing by incorporating plasmonic nanomaterials offers new opportunities to improve the sensing performance at ambient conditions [6]. In spite of these advantages, another major challenge which plasmonic metals/ZnO-based gas sensors face is their poor selectivity. For instance, these sensors may generate false responses when detecting VOCs in the presence of water (H_2_O) vapor [7]. Very few of these reported plasmonic noble metals/ZnO-based gas sensors exhibited satisfactory selectivity performance [8]. How to improve the selectivity performance of these gas sensors remains challenging [9, 10].

Recently, metal organic frameworks (MOFs), as a class of crystalline porous polymers, have attracted intensive attention due to their ultra large surface areas, and tunable porous structures and surface chemistry [11, 12]. Particularly, the selective gas adsorption behavior makes MOFs very attractive for large capacity and highly selective gas adsorption [7]. However, MOFs usually exhibit very low electrical conductivity, which limits their use in resistive sensing [13]. It is thus highly desirable to design a composite structure that takes advantages of the sensitivity of semiconductors and selectivity of MOFs. For example, Wee et al. reported a spontaneous transformation of zeolitic imidazolate framework (ZIF)-8 into a composite of ZnO nanorods and embedded in ZIF-8 matrix, which demonstrated an improved photocatalytic activity as compared to the isolated counterparts [14].

**Fig. 1:**
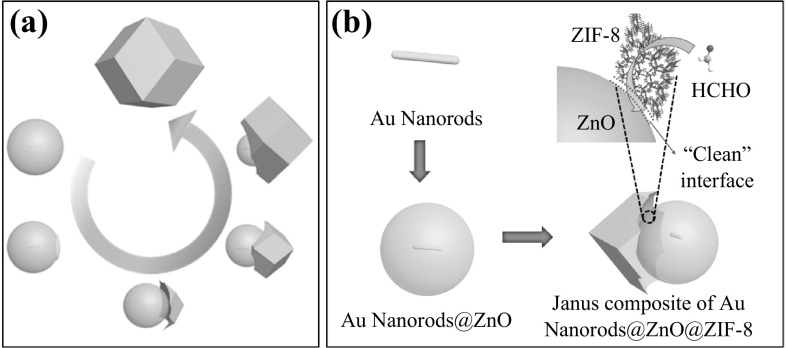
**a** Schematic illustration for the anisotropic synthesis of Au NRs@ZnO@ZIF-8, with the selective detection of HCHO highlighted in **b**

In this paper, we demonstrate that a Janus structure composed of metal oxides partially coated with MOFs can possess both desired features. Specifically, an anisotropic growth of ZIF-8 on ZnO is reported, which was realized by manipulating the reaction kinetics to control the amount of ZIF-8 nucleation sites on ZnO surface. This synthetic strategy enables us to tune the composite ratio between ZnO and ZIF-8 in a moon-phase-like manner (Fig. 1a), and to produce ZnO@ZIF-8 Janus structures. The contact area between ZnO and ZIF-8 is minimized, which later provides a “clean” interface by calcination for gas sensing (Fig. 1b). By further incorporating the plasmonic gold nanorods (Au NRs), we investigated the VOCs detection performance of the composite structure at room temperature. Systematic experimental and theoretical analyses confirmed that the Janus structure could enhance the adsorption, sensing sensitivity, and selectivity of HCHO. It could be used as a model VOC (Fig. 1b). Notably, HCHO is partially oxidized simultaneously to be non-toxic and value-added formic acid in this process, implying the potential of this nanomaterial as a medium for simultaneous detection and degradation of volatile organic compounds.

## Methods

### Chemicals and Materials

Sodium borohydride (NaBH_4_¸ 99%), silver nitrate (AgNO_3_, 99%), L-ascorbic acid (AA, 99%), chloroauric acid trihydrate (HAuCl_4_·3H_2_O, 99.9%), hexamethylenetetramine (HMT, 99%), zinc nitrate hexahydrate (Zn(NO_3_)_2_·6H_2_O, 98%), and hexadecyltrimethyl-ammonium bromide (CTAB, 98%) were purchased from Sigma-Aldrich. *N, N*-dimethylformamide (DMF) was obtained from VWR Corporation. Toluene (99.5%), and 2-methylimidazole (HMIM, 99%) was purchased from Acros Organics. Ethanol (190 proof, EtOH) was obtained from Gold Shield. Deionized (DI) water was used to prepare solutions. All chemicals were used as received without further purification. Formaldehyde gas (HCHO, 99.99%) and dry air were received from Antai Gas (Qingdao, China). Helium gas (He, purity > 99.995%) was received from Praxair.

### Preparation of Au Nanorods

Au NRs were synthesized according to the method reported by El-Sayed et al. [15]. Specifically, a seed solution was prepared by injecting NaBH_4_ solution (0.6 mL, 0.01 M) into the mixed solution of CTAB (5 mL, 0.2 M) and HAuCl_4_ (5 mL, 0.01 M), which was kept at room temperature for 30 min. A growth solution was then prepared by mixing CTAB (40 mL, 0.2 M) with AgNO_3_ (0.4 mL, 0.004 M), which was followed by adding HAuCl_4_ (40 mL, 0.001 M). AA (0.56 mL, 0.0788 M) was then added, changing the solution color from dark yellow to colorless. Upon the injection of the seed solution (0.96 mL) into the growth solution, Au NRs were obtained after aging for 15 min. Au NRs were washed by DI water for two times and finally dispersed in 10 mL of water.

### Preparation of Au@ZnO

Au NRs were further coated with ZnO by following a previous report with some modifications [16]. 0.1312 g of CTAB, 0.1071 g of Zn(NO_3_)_2_·6H_2_O, and 0.0504 g of HMT were firstly mixed with 60 mL of water. With the addition of Au NRs solution (5 mL), the mixture was stored at 80 °C for 8 h. The product was collected by centrifugation and finally dispersed in 10 mL of DMF.

### Preparation of Au@ZnO@ZIF-8

The above Au@ZnO solution was added into the mixed solution of DMF (125 mL), H_2_O (25 mL), and HMIM (0.03 M) and was aged at 65 °C. By varying the aging time, products at different stages were collected for characterization. These samples were annealed at 300 °C for 2 h to crystallize ZnO for gas sensing testing.

### Material Characterization

The structure of the samples was analyzed by a JEOL JEM-1230 transmission electron microscope (TEM). The crystallinity was characterized by a PANalytical X’Pert Pro MPD X-ray diffractometer (XRD) equipped with a Cu-*K*α radiation source (*λ* = 1.5401 Å). UV–visible spectra were measured on a Thermo Scientific Evolution 220. *In situ* diffuse reflectance infrared Fourier transform spectroscopy (DRIFTS) analysis of HCHO adsorption was conducted in Nicolet iS50 (Thermos Scientific), which is equipped with a Praying Mantis™ diffuse reflection accessory (Harrick Scientific) and a catalysis chamber. During the DRIFTS analysis, gaseous HCHO was introduced into the chamber by being purged with He gas (4 mL min^−1^). Brunauer–Emmett–Teller (BET) surface area was analyzed with an accelerated surface area and porosimetry system (ASAP 2020, Micromeritics).

### Gas Sensing Analysis

To assemble the sensor device, the samples obtained were painted on Au substrates (15 × 15 mm^2^) and then dried at 60 °C overnight. All the devices were tested under the irradiation of a 300 W Xe arc lamp (Philips) with a UV cutoff filter (*λ* ≥ 400 nm). Toluene and water were mixed with HCHO by being bubbled with dry air. The coefficient of variation (CV) was applied to evaluate the effect of interferes on the detection of HCHO [7]. The concentrations of HCHO, toluene, and relative humidity (RH) were tuned by regulating mass flow controllers. The device was kept inside a chamber with a quartz window for sensing measurements. The total gas flow was 500 mL min^−1^. The bias on the devices was 5 V, and the current was recorded with a Keithley 2401 Sourcemeter.

### Density Functional Theory (DFT) Calculations

To estimate the binding energy between the HCHO molecule and the ZnO surface and the adsorption energy of the HCHO in ZIF-8, DFT calculations were carried out using the Perdew–Burke–Ernzerhof form for the generalized gradient approximation to exchange–correlation functional [17], as implemented in the VASP code [18]. The projector augmented wave [19] method is employed to treat the valence electrons. The cutoff energy, energy convergence, and force convergence are set to 400 eV, 10^−5^ eV, and 0.005 eV Å^−1^, respectively. The ZnO (0001) surface is simulated by taking a four-layer (3 × 3) Zn-terminated slab with the bottom two layers fixed. The dangling bonds at the bottom layer are saturated by pseudo-hydrogen atoms fractionally charged with 0.5 *e* ^–^. Vacuum space of 15 Å is used along non-periodic directions. The Monkhorst–Pack *k*-mesh figure [20] is used to integrate in the reciprocal space with a *k*-grid mesh of ~ 2π × 0.02 Å^−1^. In order to incorporate long-range van der Waals interaction, the Grimme’s DFT-D2 dispersion potential [21] is also included. The HCHO molecule is adsorbed on the organic linker of ZIF-8 in the calculation.

### Finite-Difference Time-Domain (FDTD) Simulation

The three-dimensional (3D) FDTD simulation was performed to calculate local electric field distributions of the Au@ZnO core/shell NPs. The NPs were placed in water. Mesh size of 0.0005 nm was used throughout the simulation. Visible light of 740 nm, which is the simulated plasmonic peak for the Au NRs, was used as the light source.

## Results and Discussion

**Fig. 2:**
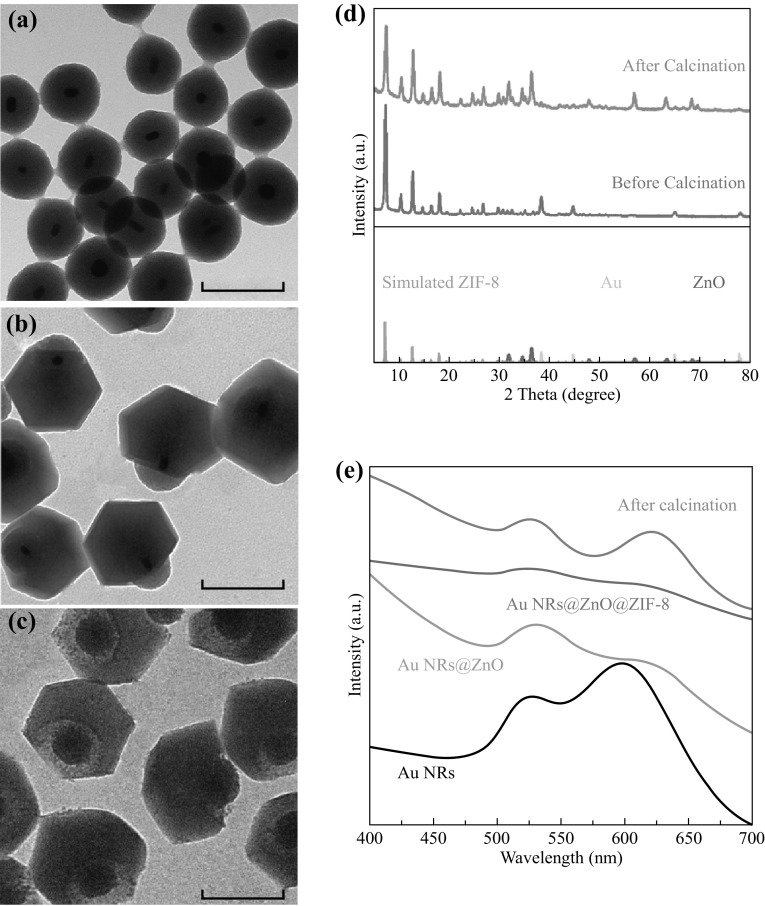
TEM images of **a** Au@ZnO core/shell NPs, **b** Au@ZnO@ZIF-8 NPs before and **c** after calcination; **d** XRD patterns of **b** and **c**; **e** UV–Vis absorption spectra of samples in **a**, **b**, **c**, and pristine Au NRs. Scale bars in **a**, **b,** and **c** represent 250, 400, and 400 nm, respectively

The synthesis process of such a Janus structure is depicted in Fig. 1b and detailed in Sect. 2. Au NRs were first obtained through a seed-mediated growth method [15]. As shown in Fig. S1, the Au NRs possess an average length of 60 nm and a diameter of 35 nm. Spherical Au@ZnO core/shell nanoparticles (NPs) with an average diameter of 180 nm were further synthesized (Fig. 2a). The pristine Au@ZnO NPs were then coated by rhombic dodecahedra after aging in DMF solution of HMIM for 40 min (Fig. 2b). XRD patterns confirmed that the rhombic dodecahedron is ZIF-8 (Fig. 2d). No peak corresponding to ZnO was detected, indicating that the ZnO was amorphous at this stage. Notably, the surface of ZnO was partially covered (Fig. 2b), implying that an anisotropic growth of ZIF-8 on ZnO took place.

To obtain crystallized ZnO for sensor applications, Au@ZnO@ZIF-8 was annealed at 300 °C for 2 h. The TEM analysis revealed that the morphology of the Au@ZnO@ZIF-8 composite after calcination was maintained to a satisfactory extent (Fig. 2c), except for the increased roughness of the surface. The XRD patterns also verified the successful crystallization of ZnO and the structural stability of the composite after calcination (Fig. 2d). In addition, the Janus particles also demonstrated satisfactory optical properties. Pristine Au NRs exhibited two plasmonic peaks at 526 and 599 nm (Fig. 2e), which can be ascribed to transverse and longitudinal resonance modes, respectively [15]. When the Au NRs were coated with amorphous ZnO, the longitudinal peak red shifted to 613 nm due to the change of the dielectric constant of the local environment. With ZIF-8 coating, the scattering effect gave a strong absorption at a shorter wavelength and weakened intensity of the plasmonic peak, due to the significant increase in particle size [22]. However, the plasmonic peaks of Au@ZnO@ZIF-8 became predominant again after calcination, which might be ascribed to the further change of the surrounding refractive index, since the guest molecules within the pores of ZIF-8 were removed and the amorphous ZnO turned to be crystallized during the calcination process.

**Fig. 3:**
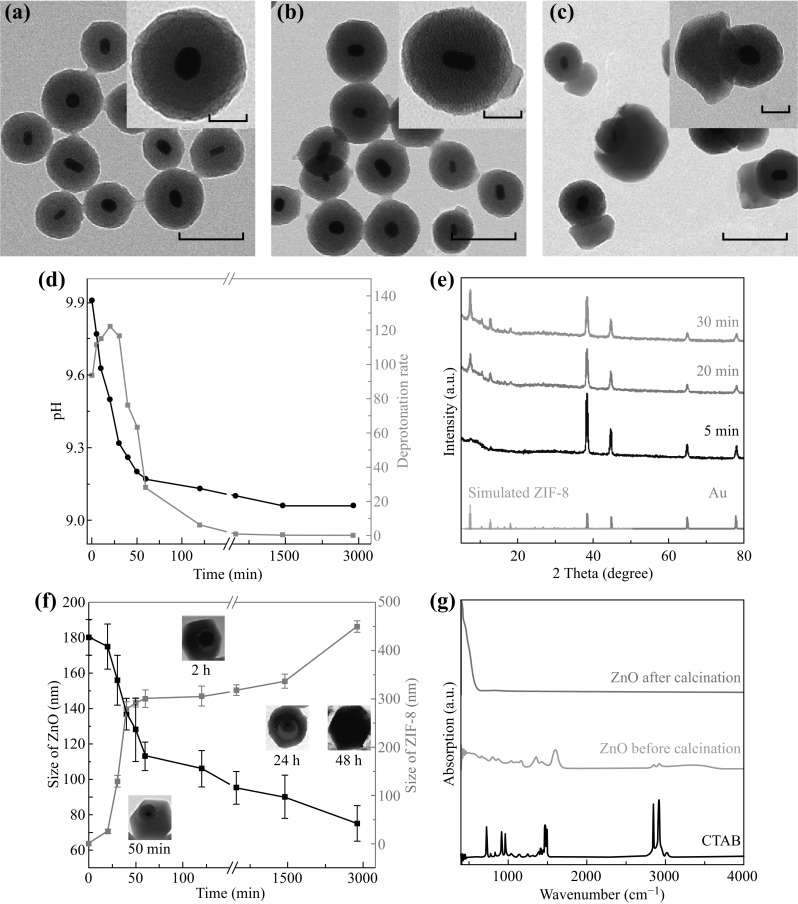
TEM images of Au@ZnO@ZIF-8 obtained at **a** 5 min, **b** 20 min, **c** 30 min; **d** pH and deprotonation rate plots versus time; **e** corresponding XRD patterns of samples in **a**, **b**, and **c**; **f** plots of Au@ZnO and Au@ZnO@ZIF-8 size versus time, insets are the represented TEM images of the products obtained at specific times; **g** FTIR absorption spectra for CTAB, Au@ZnO before calcination and after calcination at 300 °C in air for 2 h. Scale bars in **a**, **b**, and **c** represent 200 nm. Scale bars in the insets represent 60 nm

To investigate the Janus structure formation pathways, we further tuned the composition ratios between ZnO and ZIF-8 by varying the synthetic durations. After aging the Au@ZnO NPs in HMIM-DMF solution at 65 °C for 5 min, no significant morphology change was observed (Fig. 3a). However, closer observation indicated the etching effect of HMIM on the ZnO surface (inset in Fig. 3a) [23], due to the dissolution of ZnO under alkaline conditions (pH = 9.91, Fig. 3d) [24]. Interestingly, after heating for 20 min, some satellite particles were formed on the ZnO surface (Fig. 3b), which was attributed to the formation of ZIF-8, as confirmed by its corresponding XRD pattern (Fig. 3e). To obtain such a Janus structure, the concentration of HMIM should be low to ensure a single-site nucleation of ZIF-8 on the ZnO surface. On the contrary, if we increased the concentration of HMIM, a multiple-site nucleation could be observed (Fig. S2).

We found that the deprotonation rate increased rapidly and reached to its maximum at 20 min (Fig. 3d), indicating a fast crystal growth process was taking place at this stage. This is also consistent with the evolution of XRD patterns, where the peaks corresponded to ZIF-8 are first observed at 20 min. The continuously released Zn^2+^ from ZnO coordinated with HMIM in the solution and formed ZIF-8 monomers, which later attached on the patches to facilitate the growth process. In this process, the patches served as nucleation sites. For the samples obtained by aging for longer times (e.g., 30 min through 48 h) as shown in Figs. 3c, f and S3, we observed a converse size change of ZnO and ZIF-8. Au@ZnO was completely encapsuled by ZIF-8 at later stages (Fig. 3f). Since CTAB is the only surfactant in the whole synthesis process, its FTIR absorption spectrum was recorded for comparison. As shown in Fig. 3f, for the as-synthesized Au@ZnO spheres, we observed several peaks of surface residues, resulted from CTAB and its derivatives. However, after calcination, the spectrum turns to be flat (750–4000 cm^−1^), indicating the formation of a “clean” surface (without any surfactants). The sharp peak at 450 cm^−1^ is ascribed to Zn–O stretching [25]. Even though it is difficult to characterize the surfactants left on the interface between ZnO and ZIF-8 directly, the FTIR results here indicate the surfactants can be removed during the calcination process. This process is thermodynamically favorable, as the ZIF-8 monomers in the solution should preferentially attach on the ZIF-8 patches to reduce its surface energy [26], which further drives the Ostwald ripening (Fig. S4). Meanwhile, we noticed that the interface between ZnO and ZIF-8 became increasingly significant during this process, possibly due to the balance between the dissolution of ZnO and the growth of ZIF-8. This interface can be further cleaned by calcination to remove surfactants and other surface ligands (Fig. 3g), and thus benefit gas sensing. The above anisotropic growth enables us to control the ZIF-8 coating on ZnO in a moon-phase-like way and to tune the composite ratios between ZnO and ZIF-8.

**Fig. 4:**
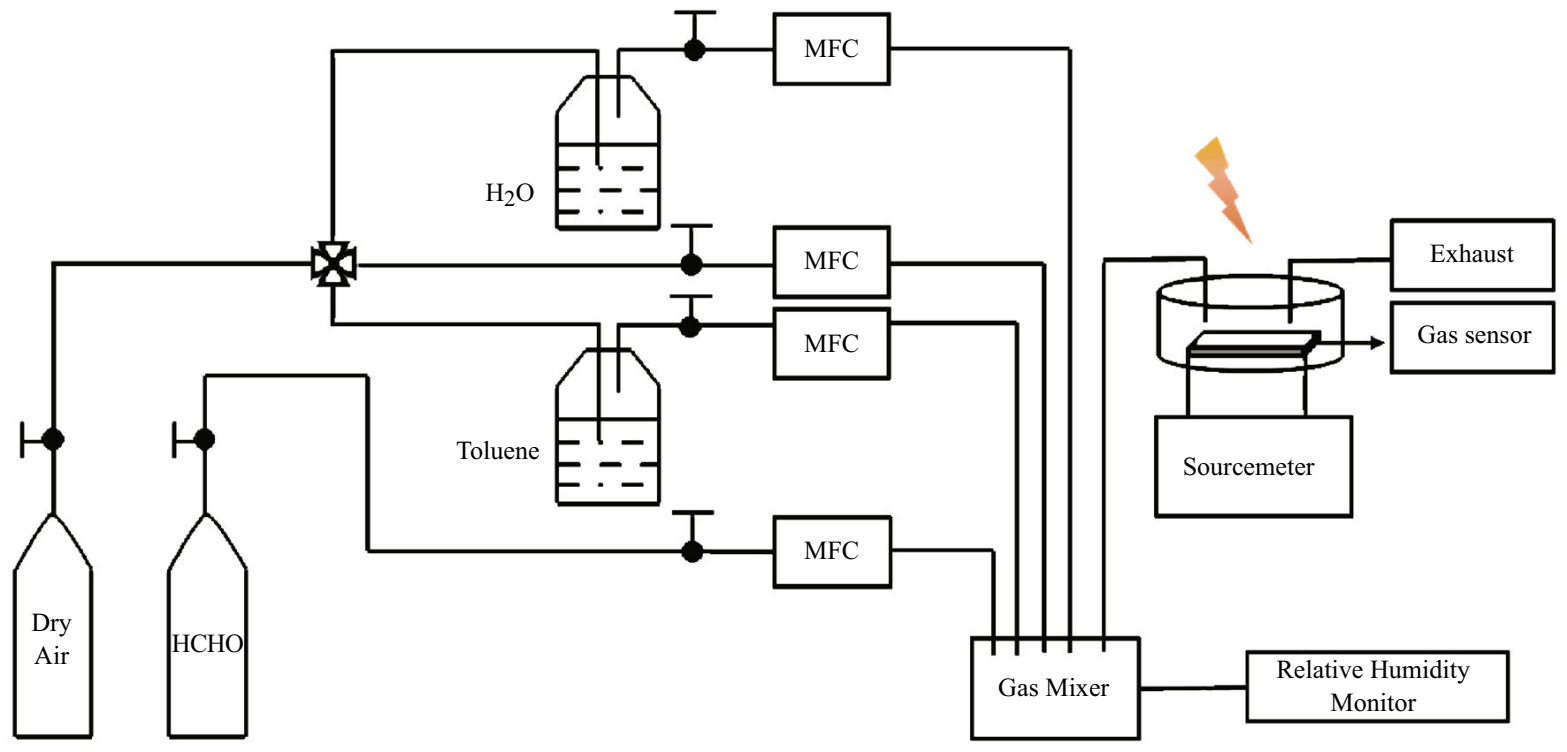
Experimental setup for HCHO detection

**Fig. 5:**
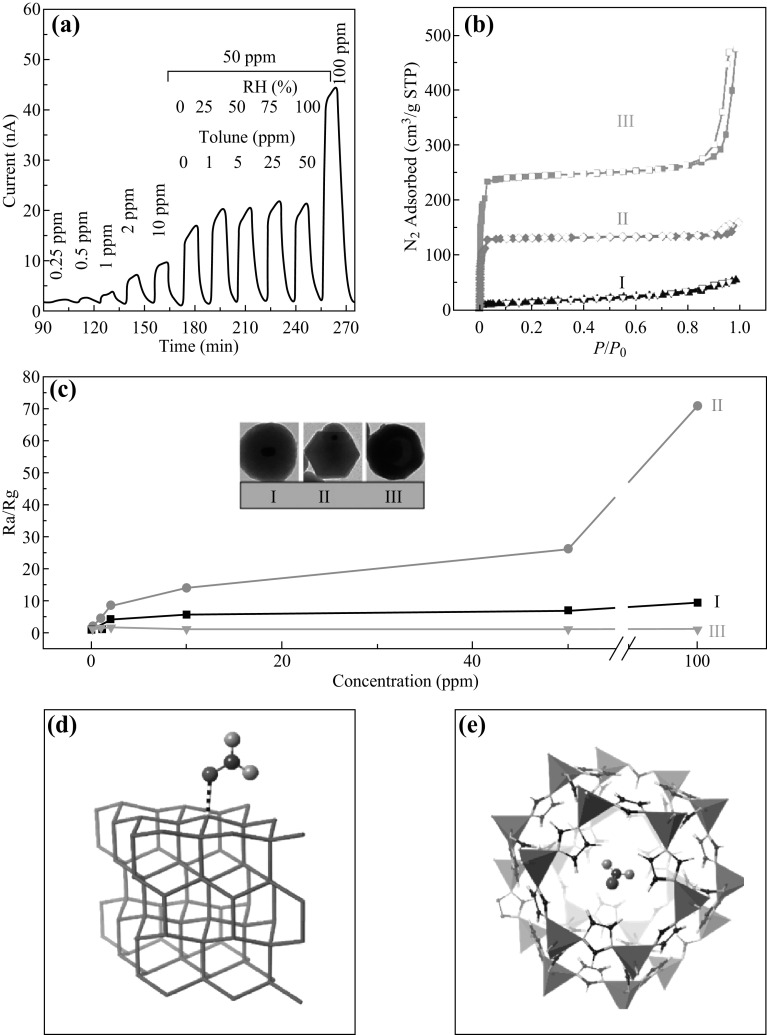
**a** Dynamic response-recovery of Au@ZnO@ZIF-8 (Sample II) to HCHO with concentrations from 0.25 to 100 ppm, **b** Nitrogen sorption isotherms for Samples I, II, and III, solid symbols represent adsorption branches, and blank symbols represent desorption branches. Sample I displays a type II isotherm, indicating its non-porosity, while with ZIF-8 coating, both Samples II and III display type I isotherm, suggesting their microporosity [28]. **c** Plots of sensor responses to HCHO concentration of different samples, inset is the typical TEM images of different samples. Optimized structures of one formaldehyde molecule adsorbed **d** on the surface of ZnO and **e** inside ZIF-8. A chemical bond is formed between the HCHO and Zn. In the case of ZIF-8, the HCHO forms a physisorption with the organic linker. Oxygen is in red, zinc in purple, carbon in gray, nitrogen in blue, and hydrogen in pink

To demonstrate the advantage of the Janus structures as gas sensors, three representative Au@ZnO@ZIF-8 samples were obtained at 0 min (i.e., pristine Au@ZnO), 40 min (Janus structure), and 48 h (complete enclosure) (denoted as Samples I, II, and III, respectively) intervals. The samples were annealed at 300 °C for 2 h and then used to detect HCHO, which is among the most concerning indoor air pollutants [27]. The schematic illustration of the sensing setup is shown in Fig. 4, and the testing procedures are detailed in **Methods**. Upon irradiation of visible light, the Janus structure demonstrated a satisfactory response-recovery over a broad range of HCHO concentrations (Fig. 5a). To highlight the selectivity performance of the Janus structure, H_2_O and toluene with varied concentrations were introduced simultaneously as interferences. As from Fig. 5a, the response of the Janus structure to HCHO remained constant, indicating its excellent sensitivity and selectivity. We further investigated the sensitivity of the three samples to various concentrations of HCHO by comparing their *R*_a_/*R*_g_, where *R*_a_ is the sensor resistance in air, and *R*_g_ is the resistance during exposure to HCHO. Pristine Au@ZnO showed a poor response to HCHO (Figs. 5c and S5). We attribute this to its lower adsorption capacity [28], since the BET surface area of pristine Au@ZnO is only 53.36 m^2^ g^−1^, much lower than that of Sample II (Fig. 5b, 500.63 m^2^ g^−1^).

The general sensing mechanism for ZnO-based sensors relies on the resistance change of ZnO when they are subjected to different environments [5]. Specifically, the near-surface region of ZnO becomes depleted of electrons due to the absorbed oxygen species. In the presence of a reducing gas (e.g., HCHO), a chemical reaction between gas molecules and oxygen species leads to electron transfer back into ZnO, thereby increasing its conductivity [29]. As a result, a higher localized concentration of HCHO near the ZnO surface should result in an enhanced signal. In the case of Sample II, ZIF-8 may “recruit” more HCHO molecules to ZnO surface [30] and thus increase the localized concentration of HCHO. Therefore, we observed an improved performance of Au@ZnO@ZIF-8 over pristine Au@ZnO. However, further increasing the ratio of ZIF-8 in the composite decreased the *R*_a_/*R*_g_ value, where almost no signal change was observed (Sample III, Figs. 5c and S5), regardless of its highest surface area (Fig. 5b, 923.75 m^2^ g^−1^). This was because the most portion of ZnO surface was covered by ZIF-8 for this sample. The conducting area between ZnO and the substrate was small, which yielded an enhanced contact resistance as compared to Sample II [31].

To get a deeper insight into the sensitivity and selectivity of ZnO covered by ZIF-8, we studied the interaction of HCHO as well as H_2_O molecules on ZnO and ZIF-8 surfaces by the DFT calculations (see **Methods** for details). For a pristine Au@ZnO, we calculated the binding energy of HCHO and H_2_O molecules. The binding energy was calculated based on Δ*E* = *E*_ZnO_ + *E*_HCHO_–*E*_HCHO-ZnO_, where *E* is the total energy. The resulting geometries are given in Fig. 5d, e. We found that the O atom of HCHO binds with the Zn atom on the ZnO surface, with a O–Zn bond length of ~ 2.1 Å. The calculated binding energy between them is found to be ~ 0.7 eV. This indicates that a chemical bond is formed between the ZnO surface and HCHO. Similarly, the H_2_O molecule also binds chemically with the ZnO surface, with a binding energy of ~ 0.6 eV and O–Zn bond length of ~ 2.2 Å. These results demonstrate that a pristine Au@ZnO is not selective as it binds to both molecules with equal strength. According to our DFT calculations, the HCHO molecules interact weakly with ZIF-8 through physisorption with a low adsorption energy ~ 0.3 eV. Thus, ZIF-8 due to its porosity will allow HCHO molecules to pass through and bind to the protected and clean ZnO surface. Consequently, when Au@ZnO is partially coated by gas-selective ZIF-8, the benefit of sensitivity and selectivity is combined. Due to stronger absorption preference of ZnO surface, an HCHO molecule will first bind to the uncovered ZnO surface, as would other molecules such as H_2_O. Thus, initially the sensitivity *R*_a_/*R*_g_ increases slowly (curve II in Fig. 5c). Once the uncovered ZnO surface is saturated, the selectivity of ZIF-8 starts to take effect; hence, one observes a sharp jump of *R*_a_/*R*_g_ in curve II.

We further investigated the selectivity to HCHO of all the three samples. The coefficient of variation (CV) was applied to evaluate the effect of interferences on the detection of HCHO [7]. The lower CV represents a better selective detection to HCHO. For Sample I, it shows a variation as high as 35.79% when relative humidity increased from 0 to 100% and toluene concentration changed from 0 to 50 ppm. However, for samples with ZIF-8 coating (II and III), they exhibit lower CVs (8.98 and 0.52%, respectively), which indicates their higher selectivity to HCHO. The higher selectivity can be attributed to the pore size of ZIF-8. Since the pore size of ZIF-8 is 3.4 Å, it is small enough to prevent toluene, which possesses a kinetic diameter of 6.7 Å, from diffusing in [32]. While the weak response of Sample III to humidity should be ascribed to the hydrophobicity of ZIF-8 [33].

**Fig. 6:**
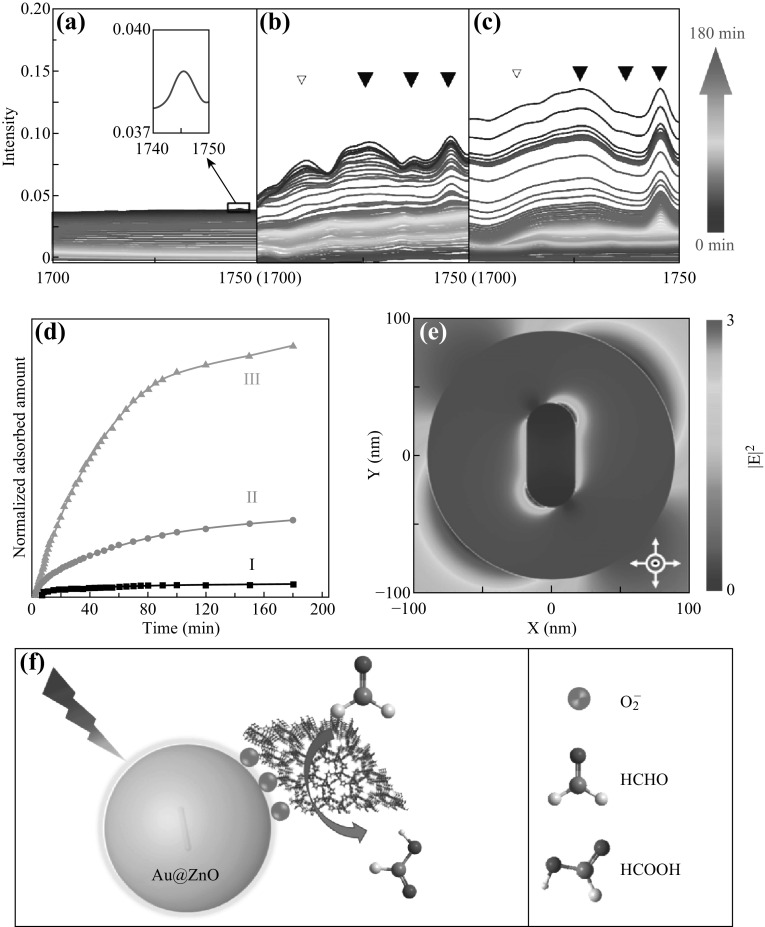
Time-resolved DRIFTS spectra on **a** Sample I, **b** Sample II, and **c** Sample III. **d** Their corresponding kinetics of HCHO adsorption (solid symbols), the blue curve represents the production kinetic of formic acid for sample II. **e** Enhancement of local electromagnetic field of Au@ZnO NPs. **f** Proposed mechanism of oxidation of HCHO into HCOOH

The sensing performance has a strong correlation with the adsorption behavior of HCHO on the materials, which was studied by time-resolved in situ DRIFTS. As shown in Fig. 6a, for Sample I, only a weak HCHO peak was observed, indicating its low absorption capacity. While for Samples II and III, the intensity of the band from 1700 to 1750 cm^−1^ increased with time elapsed. Peaks at 1726, 1736, and 1745 cm^−1^ (referred as solid triangles in Fig. 6b, **c**) can be assigned to gaseous HCHO [34], indicating the increasing amount of HCHO adsorbed on Au@ZnO@ZIF-8. As expected, HCHO adsorption amount on Sample III was even larger than that on Sample II (Fig. 6c). More obvious differences can be visualized by plotting their adsorption kinetics (Fig. 6d). Meanwhile, we also observed an intense peak at 1712 cm^−1^ for Sample II, which was assigned to formic acid (HCOOH) [34]. Since adsorbed O_2_^−^ ubiquitously exists on the surface of ZnO [35, 36], a possible reaction may happen and produce HCOOH,

1 $${\text{HCHO}} + {\text{O}}_{2}^{ - } \to 2{\text{HCOOH}} + {\text{e}}^{ - } { }$$

Our DFT calculation indicates that this reaction is most likely to happen since it is exothermic by 4.86 eV. However, the peak at 1712 cm^−1^ was less intense for Samples I and III than that for Sample II. For Sample I, this was because significantly less HCHO was adsorbed on the surface of ZnO. For Sample III, even though more HCHO was adsorbed by ZIF-8 improving its concentration near the surface of ZnO, the produced HCOOH may be difficult to detect since ZnO was encapsulated completely. Notably, we also observed a constant production of HCOOH by the Janus structure in its DRIFTS spectra (Fig. 6b), and the peak of produced HCOOH molecules is as high as that of adsorbed HCHO, implying the simultaneous oxidation of HCHO during this detection process. Since HCOOH is non-toxic and is readily metabolized and eliminated by body [37], this sensor also serves as an “air cleaner.”

Regarding the room-temperature sensing performance of the Au@ZnO@ZIF-8 Janus structure, we attributed this to the plasmonic resonance of Au NRs. Our FDTD simulation of Au@ZnO verifies an enhanced electromagnetic field near ZnO surface upon visible light irradiation (Fig. 6e), which could enhance the production of charge carriers [38, 39]. A control experiment with ZnO@ZIF-8 without Au NRs for sensing test only gave a poor response (Fig. S6), further proving that Au NRs helped improve the generation of charge carriers under visible light irradiation. Consequently, we propose a sensing mechanism as follows: The photo-generated *h* ^+^ interacts with the adsorbed oxygen ion (O_2_^−^) on the surface of ZnO [40]. Meanwhile, additional O_2_^−^ is created due to the reaction between photo-generated *e* ^−^ and ambient oxygen molecules [35]. The photo-generated O_2_^−^ then reacts with HCHO to produce HCOOH (Eq. 1). A simplified illustration of this process is shown in Fig. 6f. The electrons released during this process yield a decrease in resistance and thus an increase in current. Because ZIF-8 can increase the localized concentration of HCHO near ZnO, the above electron release process is facilitated for samples with ZIF-8 coating. Therefore, an improved performance of Au@ZnO@ZIF-8 was observed as compared to that of pristine Au@ZnO.

## Conclusion

In summary, we have developed a dual-functional system for simultaneous detection and removal of formaldehyde. This system is based on a Janus nanostructure which is composed by Au NRs, ZnO nanospheres, and ZIF-8 nanocrsytals. By manipulating the growth process, we can tune the composite ratio between ZnO and ZIF-8 and manipulate the coverage of ZIF-8 on ZnO. The Au@ZnO@ZIF-8 Janus nanostructure exhibited an impressive performance of selective detection and oxidation of HCHO, where Au NRs helped to generate charge carriers on ZnO surface under visible light irradiation. The exposed part of ZnO maintained the conductivity and photocatalytic activity of the composite, while ZIF-8 improved the adsorption capacity of HCHO and prevented the diffusion of H_2_O and toluene significantly. This proposed system may benefit the development of technologies for simultaneous detection and removal of various air pollutants. We also expect that by varying metal oxides, different MOFs can be implanted and form their corresponding Janus structures, which may advance catalysis, sensing, and other applications.

## Electronic Supplementary Material

Below is the link to the electronic supplementary material.

Supplementary material 1 (PDF 931 kb)
