# Supplementary material for: Simultaneous Detection and Removal of Formaldehyde at Room Temperature: Janus Au@ZnO@ZIF-8 Nanoparticles
Source: Nanomicro Lett. 2017 Oct 9;10(1):4. doi: 10.1007/s40820-017-0158-0 (PMC6199048; doi:10.1007/s40820-017-0158-0)
Supplement: Supplementary file 1 — Supplementary material 1 (PDF 931 kb) [file 40820_2017_158_MOESM1_ESM.pdf]

Supporting Information for

Simultaneous Detection and Removal of Formaldehyde at Room Temperature: Janus Au@ZnO@ZIF-8 Nanoparticles

Dawei Wang[1], Zhiwei Li[4], Jian Zhou[3], Hong Fang[3], Xiang He[1], Puru Jena[3], Jing-Bin Zeng[2,*], Wei-Ning Wang[1,*]

[1]Department of Mechanical & Nuclear Engineering, Virginia Commonwealth University, Richmond, Virginia 23219, United States

[2]College of Science, China University of Petroleum (East China), Qingdao 266580, People's Republic of China

[3]Department of Physics, Virginia Commonwealth University, Richmond, Virginia 23284, United States

[4]Department of Chemistry, University of California, Riverside, CA92521, United States

*Corresponding authors. E-mail: wnwang@vcu.edu, xmuzjb@163.com

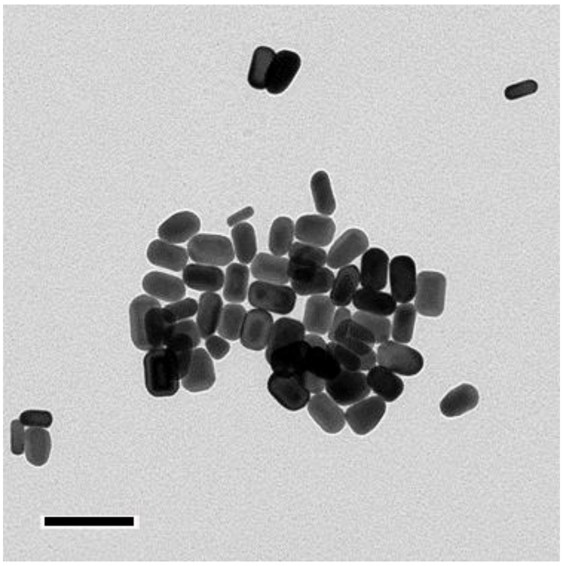

Fig. S1 TEM image of Au NRs. Scale bar represents 100 nm

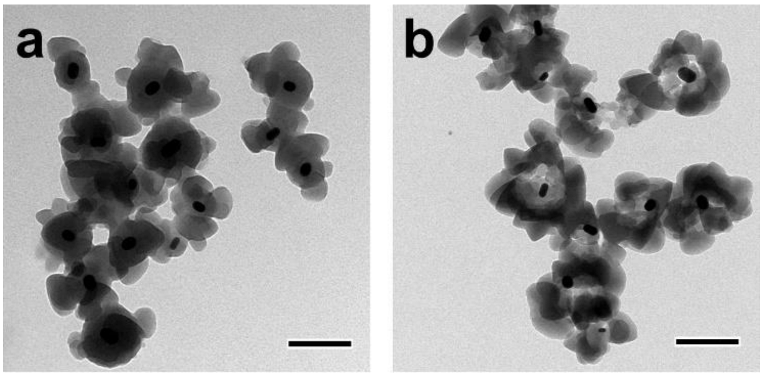

Fig. S2 TEM image of Au NRs@ZnO@ZIF-8 obtained by increasing the concentration of HMIM from 0.03 M (the samples shown in main text) to 0.3 M, at **a** 5 min and **b** 2 h. Scale bars in **a** and **b** represent 250 nm

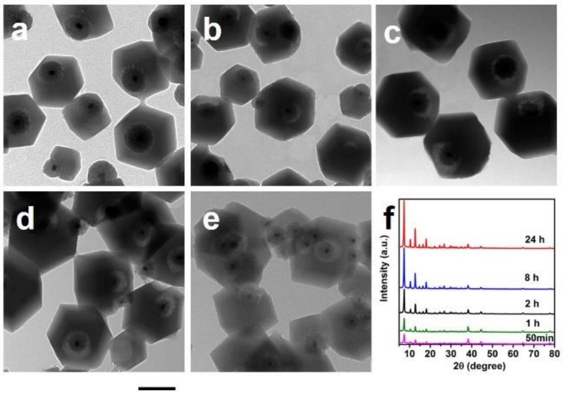

Fig. S3 TEM image of Au NRs@ZnO@ZIF-8 obtained at **a** 50 min, **b** 1 h, **c** 2 h, **d** 8 h, and **e** 24 h, **f** XRD patterns for these five stages. Scale bar represents 300 nm

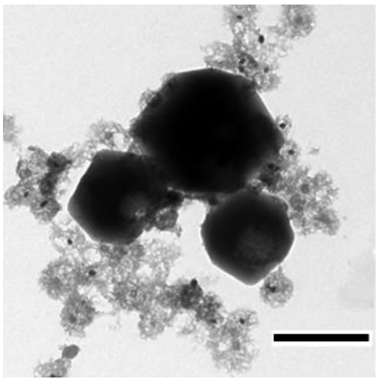

Fig. S4 TEM image of Au@ZnO@ZIF-8 obtained at 48 h; scale bar represents 400 nm. ZIF-8 which once coated on ZnO disappeared and transformed into larger crystals, which could be one of the most visual evidences for the Ostwald ripening during the ZIF-8 growth process, especially considering that the deprotonation rate is 0 at this stage (i.e., no more coordination between Zn^{2+} and HMIM)

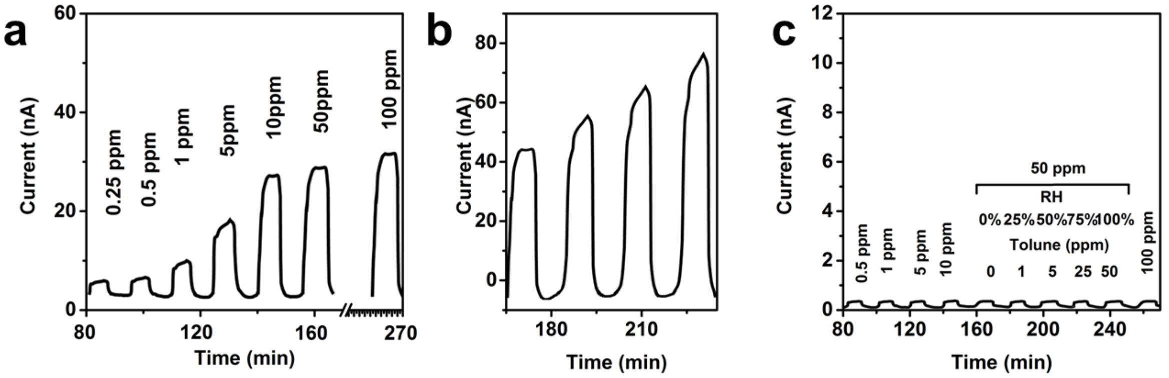

Fig. S5 Dynamic response-recovery of **a** Sample I to HCHO and **b** its selectivity performance to interferes (RH from 0 to 100%, toluene concentration from 1 to 50 ppm), **c** Sample III

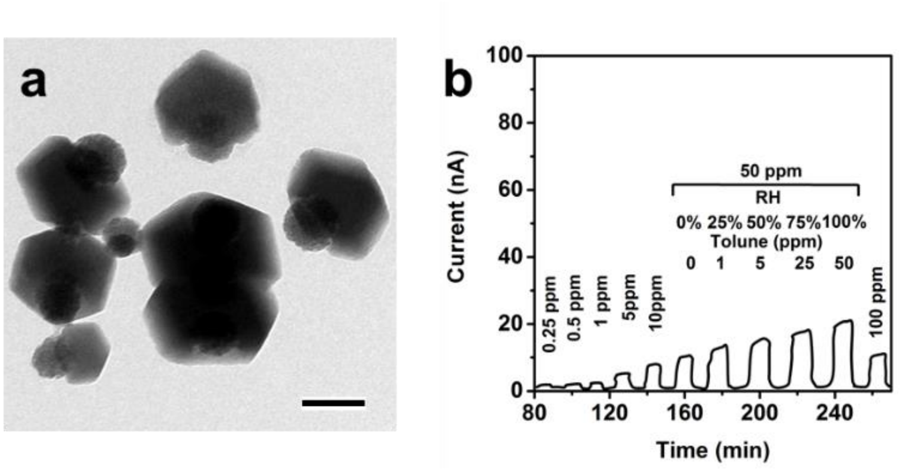

Fig. S6 a TEM image of ZnO@ZIF-8 (without Au NRs) which was synthesized under the same conditions with Sample II, and **b** its sensing performance to HCHO. Scale bar represents 200 nm.
